# Artificial Intelligence in Academic Writing

**DOI:** 10.31662/jmaj.2024-0224

**Published:** 2024-12-20

**Authors:** Soichiro Saeki

**Affiliations:** 1 Emergency Medicine and Critical Care, Center Hospital of the National Center for Global Health and Medicine, Tokyo, Japan; 2 Division of Public Health, Department of Social Medicine, Graduate School of Medicine, Osaka University, Osaka, Japan

**Keywords:** academic writing, medical education, artificial intelligence, AI, postgraduate education, undergraduate education, Japan

The author thanks dos Santos ^(1)^ for their comments on our previous manuscript ^(2)^. dos Santos discusses hyposkillia and underlines the importance of including senior preceptors in medical student education to provide clinical findings as stimulants for higher-quality articles ^(1)^. Hyposkillia is described as a “deficiency of clinical skills” that weakens the relevance of physical examination and other clinical skills in comparison to “high-tech” medicine, such as laboratory and image tests ^(3)^. Clinical skills can only be learned from patients, and this hands-on instruction is best delivered by experienced preceptors.

Similar context would apply to medical writing, which has vastly changed owing to various technologies supporting writers. Artificial intelligence (AI), in particular, supports authors in various aspects, thereby speeding up the writing process. Authors can benefit from the AI support for searching background information for manuscript writing ^(4)^. Consequently, the author has utilized AI for primary language editing in previous manuscripts ^(2), (5)^ presented in the *JMA Journal*. Language editing using AI offers several benefits: first, it is fast because it takes less than a few minutes to edit a manuscript, such as this letter. Second, it is economically feasible. Many AI services are partially free, which is a remarkable advantage for early researchers.

However, AI can introduce “hyposkillia” in academic writing. Using AI to search for background material may deter researchers from conducting research. Although finding relevant information can be difficult, the process of obtaining proper articles can disclose several crucial facts, such as patterns in current research practice, and other pertinent information that the researcher(s) may have overlooked previously. Moreover, relying only on AI can impede language growth. Language abilities are preserved and improved through AI; however, its application may limit such processing.

Furthermore, several factors must be considered while utilizing AI for research. Several AI systems “hallucinate” and generate scientifically unsound statements. Furthermore, the injudicious use of AI may result in inadvertent repetition of previous manuscripts. These issues are difficult to identify for early researchers.

Therefore, the value of preceptors is undeniable, particularly while using AI. Furthermore, as medical technology advances, hyposkillia may become prevalent in various sectors. As a young researcher and physician, the author hopes that adequate educational opportunities can be provided to passionate colleagues across Japan.

## Article Information

### Conflicts of Interest

None

### Acknowledgement

The author thanks his colleagues for helpful discussions. The author acknowledges the use of Paperpal (Cactus Communications Services Pte Ltd, Singapore) for primary language editing. The views expressed in this manuscript are of the author and do not necessarily represent the author’s institutions.

### Author Contributions

The author is solely responsible for the manuscript content. Artificial intelligence technology was used for language editing, and such content was reviewed by the author. The author’s institution played no role in the conceptualization of this manuscript.

### Approval by Institutional Review Board (IRB)

Not applicable.

## References

[1] dos Santos VM. Continuous writing, reviewing, and editing by physicians. JMA J. 2024;7(4):655-6. 39513058 10.31662/jmaj.2024-0017 PMC11543337

[2] Saeki S. Continuous writing, reviewing, and editing by physicians. JMA J. 2024;7(1):136-7. 38314415 10.31662/jmaj.2023-0115 PMC10834179

[3] Fred HL. Hyposkillia: deficiency of clinical skills. Tex Heart Inst J. 2005;32(3):255-7. 16392201 PMC1336689

[4] Salvagno M, Taccone FS, Gerli AG. Can artificial intelligence help for scientific writing? Crit Care. 2023;27(1):75. 36841840 10.1186/s13054-023-04380-2 PMC9960412

[5] Saeki S, Okada R, Horiguchi W. Promoting research under the work reform for physicians. JMA J. 2024;7(3):457-8. 39114610 10.31662/jmaj.2024-0066 PMC11301013

